# A suitable time point for quantifying the radiochemical purity of ^225^Ac-labeled radiopharmaceuticals

**DOI:** 10.1186/s41181-021-00151-y

**Published:** 2021-12-20

**Authors:** James M. Kelly, Alejandro Amor-Coarasa, Elizabeth Sweeney, Justin J. Wilson, Patrick W. Causey, John W. Babich

**Affiliations:** 1 Department of Radiology, Molecular Imaging Innovations Institute (MI3), Weill Cornell Medicine, New York, NY 10065 USA; 2 Department of Radiology, Albert Einstein College of Medicine, Bronx, NY 10461 USA; 3 Division of Biostatistics, Department of Population Health Sciences, Weill Cornell Medicine, New York, NY 10065 USA; 4 Department of Chemistry and Chemical Biology, Cornell University, Ithaca, NY 14853 USA; 5 Canadian Nuclear Laboratories, Chalk River, ON K0J 1J0 Canada; 6 Sandra and Edward Meyer Cancer Center, Weill Cornell Medicine, New York, NY 10065 USA; 7 Department of Radiology, Citigroup Biomedical Imaging Center, Weill Cornell Medicine, Belfer Research Building, Room 1600, 413 E 69th St, New York, NY 10021 USA

**Keywords:** Targeted alpha therapy, Ac-225, Radiopharmacy, Quality control

## Abstract

**Background:**

As ^225^Ac-labeled radiopharmaceuticals continue to show promise as targeted alpha therapeutics, there is a growing need to standardize quality control (QC) testing procedures. The determination of radiochemical purity (RCP) is an essential QC test. A significant obstacle to RCP testing is the disruption of the secular equilibrium between actinium-225 and its daughter radionuclides during labeling and QC testing. In order to accelerate translation of actinium-225 targeted alpha therapy, we aimed to determine the earliest time point at which the RCP of an ^225^Ac-labeled radiopharmaceutical can be accurately quantified.

**Results:**

Six ligands were conjugated to macrocyclic metal chelators and labeled with actinium-225 under conditions designed to generate diverse incorporation yields. RCP was determined by radio thin layer chromatography (radioTLC) followed by exposure of the TLC plate on a phosphor screen either 0.5, 2, 3.5, 5, 6.5, or 26 h after the plate was developed. The dataset was used to create models for predicting the true RCP for any pre-equilibrium measurement taken at an early time point. The 585 TLC measurements span RCP values of 1.8–99.5%. The statistical model created from these data predicted an independent data set with high accuracy. Predictions made at 0.5 h are more uncertain than predictions made at later time points. This is primarily due to the decay of bismuth-213. A measurement of RCP > 90% at 2 h predicts a true RCP > 97% and guarantees that RCP will exceed 90% after secular equilibrium is reached. These findings were independently validated using NaI(Tl) scintillation counting and high resolution gamma spectroscopy on a smaller set of samples with 10% ≤ RCP ≤ 100%.

**Conclusions:**

RCP of ^225^Ac-labeled radiopharmaceuticals can be quantified with acceptable accuracy at least 2 h after radioTLC using various methods of quantifying particle emissions. This time point best balances the need to accurately quantify RCP with the need to safely release the batch as quickly as possible.

**Supplementary Information:**

The online version contains supplementary material available at 10.1186/s41181-021-00151-y.

## Introduction

Targeted alpha-particle therapy (TAT) shows great promise in the treatment of cancer most recently exemplified by the successful introduction of ^223^RaCl_2_ (Xofigo®) for the treatment of skeletal metastases (Nilsson et al. 2005, 2012, 2016). Recently, TAT using astatine-211 (Zalutsky et al. 2008; Hallqvist et al. 2019), lead-212 (Meredith et al. 2018), bismuth-213 (Rosenblat et al. 2010; Cordier et al. 2010; Kratochwil et al. 2014), and actinium-225 (Juric et al. 2016; Kratochwil et al. 2016) has been investigated for the treatment of multiple cancers. Actinium-225 decays through six daughter radionuclides to stable bismuth-209 by emission of four alpha particles and two beta particles with a half-life of 9.92 days. The high yield of alpha particles per decay (Scheinberg and McDevit 2011; Morgenstern et al. 2018) and the growing supply (Robertson et al. 2018) contribute to an increasing number of investigators exploring the use of actinium-225 for TAT. Clinical evaluations of [^225^Ac]Ac-DOTATOC (Kratochwil et al. 2015), [^225^Ac]Ac-PSMA-617 (Kratochwil et al. 2018; Sathekge et al. 2019; Khreish et al. 2020), and [^225^Ac]Ac-DOTA-Substance P (Krolicki et al. 2019) report significantly improved responses in patients with neuroendocrine tumors, prostate cancer, and glioma, respectively. Dramatic responses are even observed in patients refractory to beta-particle therapy (Kratochwil et al. 2016). In addition to these small molecule radioligands, [^225^Ac]Ac-J591 is currently in Phase I clinical trials for radioimmunotherapy of prostate cancer (Tagawa et al. 2018). Given these early and promising findings clinical investigations using actinium-225 TAT will likely continue to grow.

Actinium-225 is introduced to tumor-targeting vectors through complexation by a chelating moiety integral to the vector. This is commonly achieved using bifunctional derivatives of 1,4,7,10-tetraazacyclododecane-1,4,7,10-tetraacetic acid (DOTA) (Kratochwil et al. 2015, 2018; Krolicki et al. 2019; McDevitt et al. 2002) conjugated to the vector, although new bifunctional macrocyclic chelators that complex actinium-225 with high specificity and stability have also been recently reported (Thiele et al. 2017; Ramogida et al. 2019; Yang et al. 2020; Li et al. 2020; Thiele and Wilson 2018). The preparation of ^225^Ac-labeled radiopharmaceuticals typically requires a period of incubation of the chelate-vector conjugate and an actinium-225 salt (e.g., [^225^Ac]AcCl_3_ or [^225^Ac]Ac(NO_3_)_3_) at acidic or neutral pH, followed by formulation for injection and sterilization of the radiopharmaceutical solution. In some cases, purification of the radiolabeled compound from uncomplexed actinium-225 may be required.

Each batch production of a radiopharmaceutical for clinical use must undergo quality control (QC) testing to meet release criteria before being dispensed to the patient. A critical release parameter is radiochemical purity (RCP); the proportion of the total radioactivity in a sample present as the desired radiolabeled species, the ^225^Ac-labeled vector. Currently, the RCP of ^225^Ac-labeled radiopharmaceuticals is primarily assessed using a form of thin-layer chromatography (TLC) and a mobile phase that effectively separates non-complexed radiometals and radiolabeled impurities from the desired radiopharmaceutical. However, the complex decay chain of actinium-225 renders quantification of RCP challenging because daughter radionuclides may not be complexed to the vector and may distribute across the chromatographic plate. Prior to secular equilibrium, the ratio of actinium-225 to its daughter radionuclides constantly changes. The daughter radionuclides may be detected differently to actinium-225, leading to inaccurate quantification of RCP. At secular equilibrium, this ratio is constant and highly accurate measurement of RCP is possible. Secular equilibrium between actinium-225 and its daughter products is reached after 20 h (Poty et al. 2018; Kruijff et al. 2019), but it is impractical to delay quantification of RCP for more than 20 h in a clinical production setting. Consequently, sites producing ^225^Ac-labeled radiopharmaceuticals currently wait between 1 and 12 h after running the TLC plate before quantifying RCP (Kratochwil et al. 2016; Ramogida et al. 2019; Shukurov et al. 2019; Deal et al. 1999;). Such a range of practices introduces variability to the radiopharmaceutical production and QC process. Ultimately, this may lead to confounding results when clinical evaluations are compared across multiple sites and potentially delay approval of the radiopharmaceutical by governing regulatory agencies.

To work toward a consensus quality control protocol for ^225^Ac-labeled radiopharmaceuticals, we aimed to determine the earliest pre-equilibrium time at which RCP can be accurately quantified. Herein we present a statistical analysis of over 500 radioTLC results performed at various time points with vectors that gave a range of radiometal complexation. Using this analysis, we compare the RCP at secular equilibrium for radiopharmaceuticals bearing either bifunctional DOTA or bifunctional *N*,*N*′-bis[(6-carboxy-2-pyridyl)methyl]-4,13-diaza-18-crown-6 (macropa) chelating moieties to the RCP at measurements taken before equilibrium is reached. Our data, supported by a statistical model derived from these data, suggest a suitable pre-equilibrium time at which the RCP of ^225^Ac-labeled radiopharmaceuticals can be quantified with sufficient accuracy to permit batch release.

## Materials and methods

### Radiolabeling experiments

In order to generate a diverse range of radiochemical yields, reactions were performed using various chelators conjugated to a targeting vector, varying the concentration of the chelator–vector conjugate, and varying reaction time and temperature. All reactions were performed in triplicate. Six small molecule ligands targeting prostate-specific membrane antigen (PSMA), RPS-072 (Kelly et al. 2019a), RPS-074 (Kelly et al. 2019b), EuK-106 (Kelly et al. 2017), EuK-107(Kelly et al. 2017), RPS-088, and RPS-092 (Additional file 1: Fig. S1), were prepared as stock solutions of 0.01–1 mg/mL in DMSO. Actinium-225 (9.25 MBq) was obtained from a thorium generator (Perron et al. 2020) at Canadian Nuclear Laboratories and supplied as the dried [^225^Ac]AcCl_3_ salt. The [^225^Ac]AcCl_3_ was dissolved in 1 mL 1 M NH_4_OAc, pH 7.0, transferred by pipette to a 50 mL centrifuge tube (Corning), and diluted to 45 mL in 1 M NH_4_OAc. One mL of the stock solution, containing approximately 205 kBq [^225^Ac]Ac(OAc)_3_, was transferred by pipette to a plastic Eppendorf tube placed on a digital ThermoMixer (Eppendorf) heating block. Then 20 µL of the ligand stock solution (0.01–1 mg/mL in DMSO) was added and the reaction was shaken at 300 rpm at either 25 °C or 95 °C (Table 1). A 3 µL aliquot of the reaction mixture was withdrawn and deposited on the origin of a silica gel 60-coated aluminum plate (Sigma Aldrich) after incubating the reaction for 1 min, 5 min, and 15 min.

**Table 1:** Reaction conditions for radiolabeling

Compound	Chelator	[Compound] (μM)	T (°C)
RPS-072	DOTA	9.0	95
RPS-074	Macropa	8.9	25
		0.89	
		0.089	
RPS-088	Macropa	10.4	25
		1.04	
		0.104	
RPS-092	Macropa	8.4	25
		0.84	
EuK-106	DOTA-106	18.2	95
EuK-107	DOTA-107	20.0	95

Conditions were selected to increase diversity rather than to maximize radiochemical yield. The chelator associated with each molecule is indicated. Full structures are provided in Additional file 1: Fig. S1

### Validation of TLC method

A TLC method was developed to separate the metal complexed ligand from uncomplexed actinium-225 and its daughter radionuclides. The reaction of 10.4 µM RPS-088 with [^225^Ac]AcCl_3_ was incubated for 15 min at 95 °C and then purified using a Sep-Pak C18 light cartridge as previously described (Ramogida et al. 2019; Kelly et al. 2019b). The radiolabeled product was eluted with 10% *v/v* EtOH/saline. A 3 µL aliquot of the elution was immediately spotted on the TLC plate and run as described below. A separate plate containing a 3 µL aliquot of a solution of [^225^Ac]Ac-EDTA was run as a control. Full details and representative phosphor images can be found in the Additional file 1: Fig. S2.

### Measurement of reaction purity

**Fig. 1:**
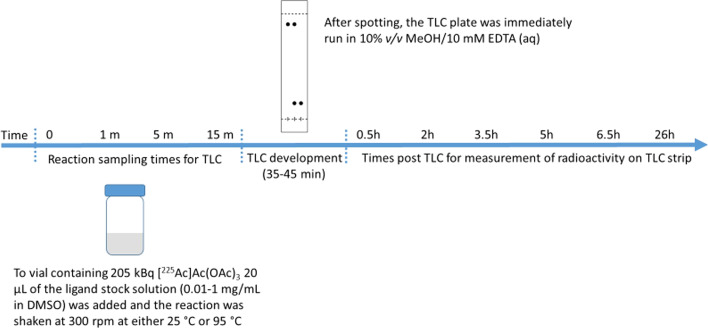
Description of the experiment and method of measuring radiochemical purity

After the final aliquot was deposited on the TLC plate, the plate was immediately run in freshly prepared mobile phase comprised of 10% *v/v* MeOH/10 mM aqueous EDTA. The plates were developed until the solvent front traveled at least 60% of the total plate length (35–45 min). Upon removal from the mobile phase, the plates were dried and then assayed by direct exposure on a phosphor screen for 2 min at 0.5 h, 2 h, 3.5 h, 5 h, 6.5 h, and 26 h using a Cyclone Plus Storage Phosphor System (Perkin Elmer) (Fig. 1).

**Fig. 2:**
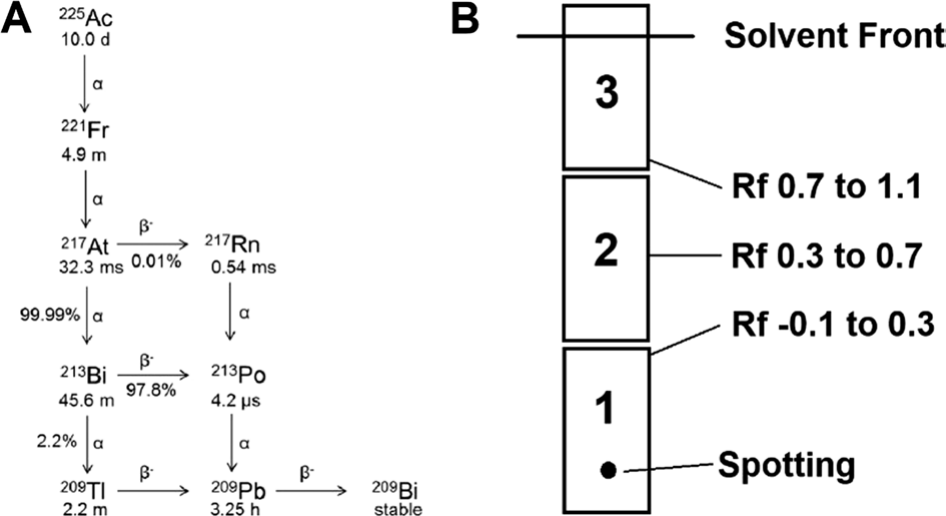
**A** Actinium-225 decay scheme. **B** General representation of the box grid used to quantify radiolabeling by radioTLC

The phosphor screen was erased with homogenous white light (5 min exposure) between assay exposures. The retention factors, R_f_, of the radiolabeled vectors were 0.0–0.3, while [^225^Ac]Ac-EDTA (and daughter radionuclides present at time of spotting) migrated mainly toward the solvent front (R_f_ > 0.7). Labeling yields were determined using OptiQuant™ software (Perkin Elmer) by fitting a boxed grid to the plate and quantifying the counts in all regions. Radiochemical yield was defined as the ratio of the counts in the box corresponding to the labeled product to the sum of the counts in all boxes (Fig. 2). Radiolabeling yield was plotted as a function of measurement time using GraphPad Prism 8 (GraphPad Software). Reactions did not undergo further purification, therefore reaction yield was equivalent to RCP.

### Statistical modeling

All statistical modeling was performed in the R environment (version 3.5.0, R Foundation for Statistical Computing, Vienna, Austria). Ninety-nine observation sets (11 initial reaction conditions × 3 reaction times × 3 replicates) were randomly assigned to training and validation sets (66 sets to the training set and 33 sets to the validation set). As the labeling percentage for the samples were bounded between 0 (0%) and 1 (100%), a generalized linear model with binomial link was fit to the data in the training set to model the relationship between the measurement at 26 h, after secular equilibrium has been reached (defined as the true RCP), the time of each observation, the yield of the observation at that time point, and the interaction between the two. Samples with a value of 0.9 or more at 26 h were weighted 10 times more than the other samples in the model in order to optimize our model for predictions of values in this area.

Predictions from the model were made in the validation set and mean absolute error (MAE) was calculated in the validation set for strata of each time (predictions using data at 0.5 h, 2 h, 3.5 h, 5 h, and 6.5 h). We also report a predicted outcomes table, where the generalized linear model was retrained using the entire dataset, continuing to weight the samples in the manner described above. The predicted RCP at 26 h from the model for each of the measured values is reported as well as 95% prediction intervals.

We fit a random forest classification model (Ho 1995; Liaw and Wiener 2002) on the training set to predict labeling percentage above a given threshold. The predictors in this model include the time that a given observation is made and the yield of that observation. Models were fit using a discrimination threshold of 0.9 (90%) or 0.95 (95%). For a threshold of 0.9, there were 140 observations ≥ 0.9 in the data set, and 190 observations < 0.9. For a threshold of 0.95, there were 125 observations ≥ 0.95 in the data set, and 205 observations < 0.95. A receiver operating characteristic (ROC) curve is plotted for each classification model in the validation set, and area under the curve (AUC) and 95% confidence interval (CI) are reported.

The model was validated using an independent data set consisting of observations made at 0.5, 1.5, 2.0, 2.5, and 26 h. The MAE of the predictions are reported at each time point.

## Results

### Radiolabeling

Mean radiolabeling yield after 1 min, as assessed 26 h after running the TLC, ranged from 2.7 ± 0.55% to 98.8 ± 0.09%. The highest radiochemical yields were obtained with the macropa-conjugates RPS-074 and RPS-088, which exceeded 97% at a ligand concentration of 9–10 μM. The range after 5 min was 3.8 ± 1.95% to 99.0 ± 0.04%, and the range after 15 min was 11.2 ± 5.25% to 99.2 ± 0.32%. RPS-074 and RPS-088 were rapidly labeled at 25 °C, and there was no significant change in labeling yield when ligand concentration decreased from approximately 10 μM to approximately 1 μM. At a ligand concentration of approximately 0.1 μM, radiolabeling yield was highly variable, ranging from 5.9% to 92.1%. EuK-107, bearing an amine analogue of the 3p-C-DEPA chelator, was rapidly labeled at 95 °C, while DOTA-containing RPS-072 reached 93.2 ± 1.21% after 15 min at 95 °C and a ligand concentration of 9 μM. In agreement with previously published results (Kelly et al. 2017), EuK-106 was labeled poorly (24.8 ± 2.36%) even after 15 min at 95 °C.

**Fig. 3:**
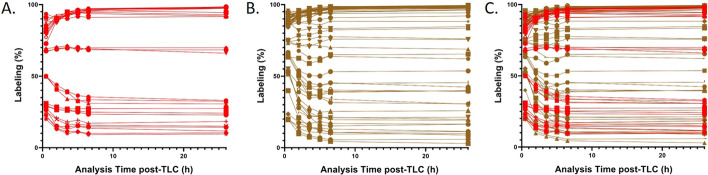
Plot of radiolabeling yield versus measurement time after TLC for multiple reaction conditions. Radiolabeling was performed using RPS-072, RPS-074, RPS-088, RPS-092, EuK-106, or EuK-107 and [^225^Ac]AcCl_3_ in 1 M NH_4_OAc. Conditions are described in Table 1. Activity distribution on the TLC plates was visualized at the specified times using a Cyclone Plus Storage Phosphor System. Quantification of labeling was performed using OptiQuant™ software. Each data point represents a unique measurement, with measurements taken from the same reaction at different time points joined by a curve. **a**DOTA-containing ligands (RPS-072, EuK-106, EuK-107). **b** Macropa-containing ligands (RPS-074, RPS-088, RPS-092). **c** All compounds

**Fig. 4:**
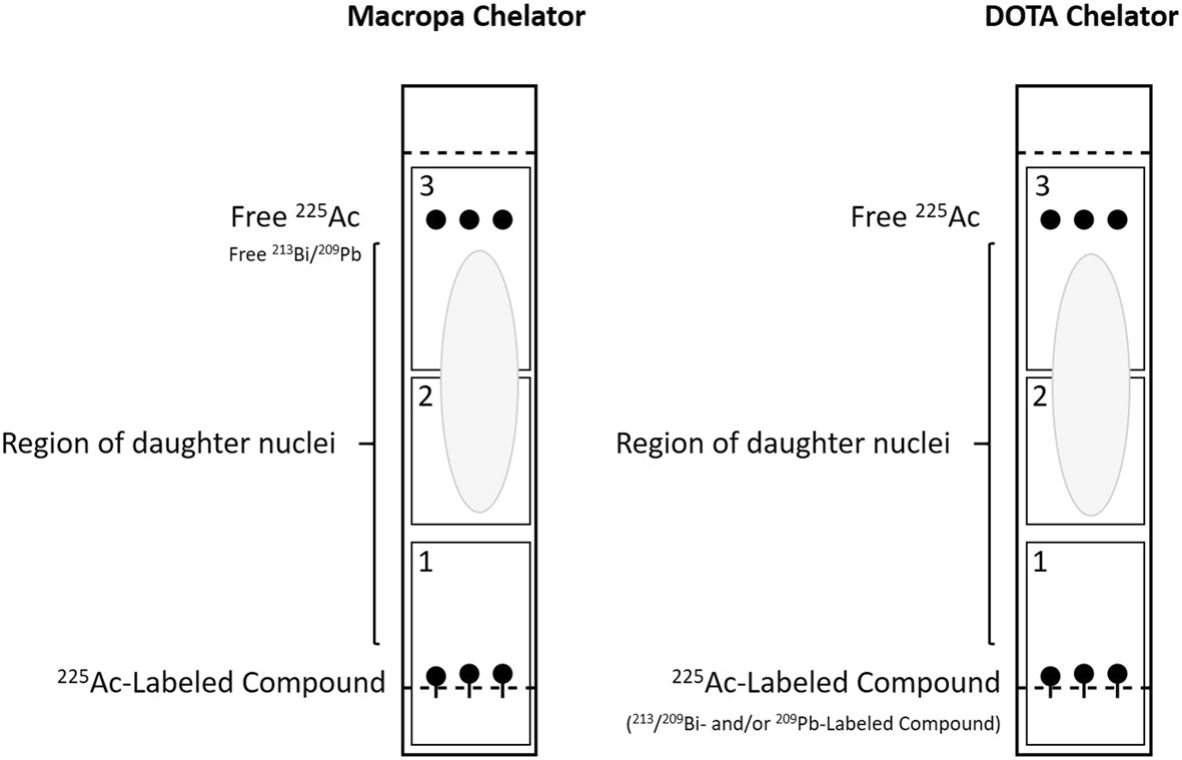
Schematic of the distribution of radiolabeled compounds, unbound metal (transformed into the EDTA complex upon exposure to the mobile phase), and daughter nuclei ejected from the labeled compounds following radioactive decay during the TLC running period. The DOTA-containing ligands are expected to chelate ^209/213^Bi and ^209^Pb in solution at 95 °C, but not to chelate daughter products generated by radioactive decay

Three clusters of radiolabeled compounds were observed (Fig. 3). For those compounds that were efficiently labeled (> 85% as determined at 26 h), quantification at early time points underestimated the true purity. This is likely due to the accumulation of daughter products in sections 2 and 3 of the TLC grid that were released from the decay of the ^225^Ac-labeled radiopharmaceutical in section 1 (Fig. 4). By contrast, the labeling of compounds with low RCY (< 40% at 26 h), is overestimated at early measurement times. This may reflect chelation of shorter-lived daughter products (predominantly ^213^Bi) in the labeling solution by the ligands. It is likely that this is particularly pronounced for the DOTA-containing ligands. Finally, the purity of compounds labeled with moderate efficiency, 50% ≤ RCY ≤ 75% at 26 h, was estimated with reasonable accuracy at early measurement times.

### Statistical modeling

**Fig. 5:**
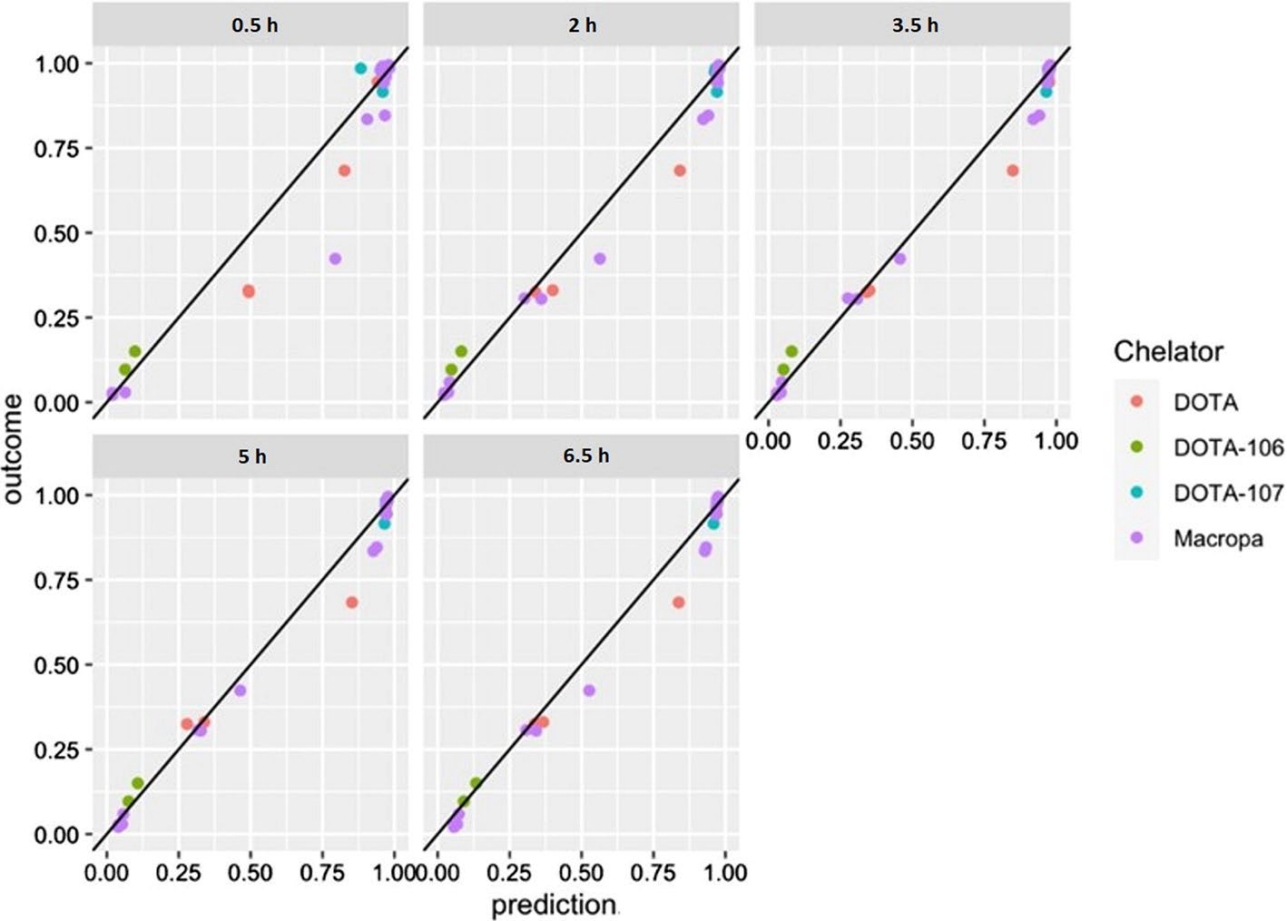
Predictive accuracy of QC measurements taken at 0.5 h, 2 h, 3.5 h, 5 h, or 6.5 h after running the radioTLC plate. Each plotted point represents the outcome of a reaction involving a compound conjugated to DOTA (red) or a DOTA-derivative (green, cyan) or a compound conjugated to macropa (purple). X-axis: Predicted radiochemical yield. Y-axis: True radiochemical yield, defined as the radiochemical yield when measured at 26 h after running the TLC

We developed a statistical model to predict the RCP at secular equilibrium (true RCP) of any RCP measured at an early pre-equilibrium time point. The predicted RCP and true RCP are well matched for reactions with high yield (RCY ≥ 85%) and low yield (RCY ≤ 25%). In the intermediate range 25% ≤ RCY ≤ 85%, predictions generally overestimate the true RCP (Fig. 5). The accuracy of the predictions is lowest at 0.5 h, at which time MAE is 0.053 (5.3%). For TLC reads performed from 2 h onwards, the accuracy of the model is high. MAE is 3.2% at 2 h, decreases to 2.6% at 3.5 h and 5 h, and 2.9% at 6 h. This indicates that labeling yield may be over- or underestimated by no more than 3% after 2 h. The analysis was also performed separately for the macropa-containing ligands RPS-074, RPS-088, and RPS-092, and for the DOTA-containing ligands RPS-072, EuK-106, and EuK-107. Neither sub-analysis differed significantly from the analysis using the entire data set (Additional file 1: Fig. S3–S5, Tables S1, S2).

The model was validated using an independent data set collected for radiolabeling of a macropa-conjugated antibody (Additional file 1: Fig. S6). The predictions of the model were highly accurate, with an MAE of 1.3% at 0.5 h that decreases to 0.9%, 0.8%, and 0.8% at 1 h, 1.5 h, and 2 h, respectively (Additional file 1: Table S3). Our model predicts that RCP in excess of 90%, as measured at any time point, is highly likely to translate to an RCP at secular equilibrium greater than 95% (Table 2). However, range of expected values at the 30 min time point for RCP ≤ 97% includes a lower bound < 95%. This means that a measurement of 97% at 30 min could possibly result in a true value < 95%. By 2 h, the range of expected values is 95–100% for all RCP ≥ 94%. This implies that a purity threshold of 95% for batch release will be definitively met if the yield is measured to be ≥ 98% at 0.5 h or ≥ 94% at 2 h after running the TLC plate. The expanded prediction intervals at the endpoints of the model reflect the absence of data for t < 0.5 h and t > 6.5 h, which results in inaccurate extrapolation.

**Table 2:** Predicted RCP, defined as the RCP measured at secular equilibrium, based on measurements of taken prior to equilibrium

RCP AT 0.5 h (%)	EXPECTED RCP (%)	RCP at 2 h (%)	EXPECTED RCP (%)	RCP at 3.5 h (%)	EXPECTED RCP (%)	RCP at 5 h (%)	EXPECTED RCP (%)	RCP at 6.5 h (%)	EXPECTED RCP (%)
0	1 (0, 23)	0	2 (0, 14)	0	2 (0, 12)	0	4 (1, 17)	0	5 (0, 39)
10	3 (0, 33)	10	4 (1, 23)	10	5 (1, 19)	10	7 (2, 26)	10	10 (1, 48)
20	6 (1, 45)	20	8 (2, 34)	20	11 (3, 30)	20	14 (4, 37)	20	17 (3, 58)
30	14 (2, 58)	30	17 (5, 48)	30	21 (8, 43)	30	24 (9, 51)	30	29 (7, 68)
40	29 (7, 70)	40	32 (12, 62)	40	36 (18, 58)	40	40 (20, 64)	40	44 (16, 76)
50	50 (19, 80)	50	52 (29, 75)	50	55 (36, 72)	50	58 (37, 76)	50	60 (31, 84)
60	71 (43, 89)	60	72 (53, 85)	60	73 (58, 84)	60	74 (57, 86)	60	75 (50, 90)
70	85 (68, 94)	70	85 (74, 92)	70	85 (76, 91)	70	85 (74, 92)	70	85 (68, 94)
80	93 (84, 98)	80	93 (87, 97)	80	93 (87, 96)	80	92 (85, 96)	80	92 (80, 97)
90	97 (92, 99)	90	97 (93, 99)	90	97 (93, 98)	90	96 (92, 98)	90	96 (88, 99)
91	97 (92, 99)	91	97 (93, 99)	91	97 (94, 98)	91	96 (92, 98)	91	96 (88, 99)
92	98 (92, 99)	92	97 (94, 99)	92	97 (94, 98)	92	97 (93, 98)	92	96 (89, 99)
93	98 (93, 99)	93	98 (94, 99)	93	97 (94, 99)	93	97 (93, 99)	93	96 (89, 99)
94	98 (93, 99)	94	98 (95, 99)	94	97 (95, 99)	94	97 (93, 99)	94	97 (90, 99)
95	98 (94, 100)	95	98 (95, 99)	95	98 (95, 99)	95	97 (94, 99)	95	97 (90, 99)
96	98 (94, 100)	96	98 (95, 99)	96	98 (95, 99)	96	97 (94, 99)	96	97 (90, 99)
97	98 (94, 100)	97	98 (95, 99)	97	98 (96, 99)	97	98 (94, 99)	97	97 (91, 99)
98	99 (95, 100)	98	98 (96, 99)	98	98 (96, 99)	98	98 (95, 99)	98	97 (91, 99)
99	99 (95, 100)	99	99 (96, 99)	99	98 (96, 99)	99	98 (95, 99)	99	98 (92, 99)
100	99 (95, 100)	100	99 (96, 100)	100	98 (96, 99)	100	98 (95, 99)	100	98 (92, 99)

Data were generated using unpurified reactions and are expressed as expected value with prediction interval

**Fig. 6:**
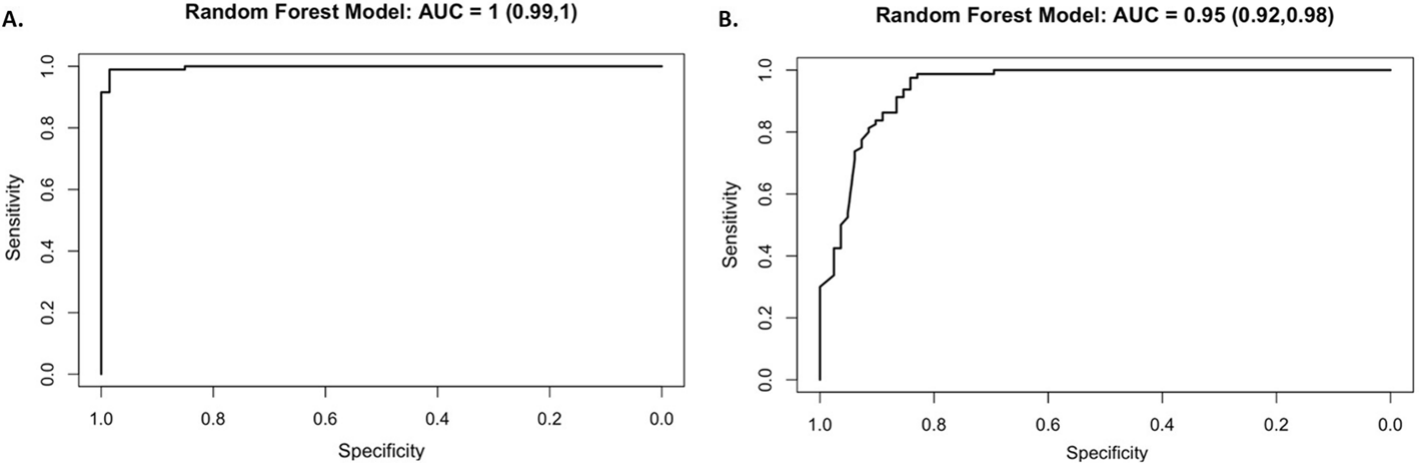
Random forest classification models describing the accuracy of statistical predictions given a discrimination threshold of **a** 0.9 or **b** 0.95

We derived two classification models to further evaluate the accuracy of our predictions. With a discrimination threshold of 0.9, which corresponds to 90% labeling yield, the predictive accuracy of the model is 99% (160/162) (Fig. 6a). Both of the incorrect predictions are made at the 0.5 h analysis time point. An increase of the discrimination threshold to 0.95 results in a slight decrease in predictive accuracy to 91% (147/162) (Fig. 6b). Much of the discrepancy between prediction and true outcome is due to “false positives” (14/162), in which the prediction overestimates the true yield. The AUC of both curves exceeds 0.95, confirming the high accuracy of the tests.

## Discussion

Despite the 9.92 d half-life of actinium-225, it is advantageous to be able to administer a ^225^Ac-labeled radiopharmaceutical as soon as possible after radiosynthesis is completed. One advantage is the simplification of logistics: the radiopharmaceutical can be prepared and administered locally during normal business hours. Ideally, the radiolabeling is performed with high efficiency, allowing the reaction mixture to be buffered and directly administered to the patient without a purification step. It is therefore advantageous to determine as quickly as possible whether an additional purification step is required to remove uncomplexed actinium-225 prior to administration of the radiopharmaceutical. Secondly, a shorter shelf-life may guarantee the radiochemical purity of the radiopharmaceutical by virtue of minimizing the number of decays that occur while the compound is in its final formulation, thereby avoiding product decomposition owing to radiolysis. Each decay of actinium-225 results in the emission of one α-particle, with simultaneous recoil of the francium-221 daughter nucleus. This alpha recoil energy is sufficient to liberate the daughter nucleus from the chelator into solution, potentially leading to the administration of non-targeted ionizing radiation to the patient (Kruijff et al. 2015). Furthermore, the α-particle itself may induce radiolytic damage to the radiopharmaceutical. If this damage occurs to the targeting moiety of the ligand, it may reduce in vivo targeting and lead to further accumulation of radioactivity in non-target tissue.

The desire to quickly release the production batch must not supersede the importance of accurately assessing the purity of the compound before administration. In the case of ^225^Ac-labeled radiopharmaceuticals, this can be done by using a form of solid state detection, such as phosphor imaging. In this scenario, all particles may be detected, and the sensitivity of the approach is enhanced. However, γ particles are detected by the phosphor screen with a different efficiency than α and β particles. In addition, the disruption of the secular equilibrium further complicates the analysis of radiochemical purity. To further complicate the analysis, an initial activity of 37 MBq actinium-225 reaches secular equilibrium with francium-221 after 55 min and with bismuth-213 after approximately 6.5 h (Tichacek et al. 2019). On this basis, we analyzed the TLC plates 6.5 h and 26 h after they were run, a time point at which actinium-225 is in secular equilibrium with all of its daughter radionuclides. We also selected four earlier time points (Additional file 1: Fig. S7), separated by 90 min intervals in order to investigate meaningfully different QC scenarios—waiting 30 min or 60 min after running the TLC may not have a major impact on release testing, but waiting 2 h rather than 30 min may be more significant. These intervals also accommodated the logistical constraints imposed by the experiments. In total, we made 585 observations spanning labeling yields from 1.8% to 99.5%. The measurements were more heavily distributed toward the extremes, with 42% of observations lying in the range 90–100%, and 21% lying in the range 0–20% (Additional file 1: Fig. S8). It is likely that the unequal distribution of measurements influenced our models. However, with protocols for radiolabeling DOTA and macropa with actinium-225 now well established, a dataset weighed heavily by yields > 90% is consistent with the typical radiopharmacy experience.

An alternative strategy to the use of phosphor imaging is to use γ-spectrometry (Kratochwil et al. 2016; Ramogida et al. 2019; Robertson et al. 2017). Our mathematical model excluded francium-221 due to its short half-life, but its decay results in a 218 keV γ-emission that is detected by both phosphor imaging and γ-spectrometry. This 218 keV γ-emission can be used to indirectly detect and quantify actinium-225(Kratochwil et al. 2016; Ramogida et al. 2019; Robertson et al. 2017). Quantification by this method is only possible after waiting at least 60 min for the equilibrium between actinium-225 and francium-221 to be reached ((Kratochwil et al. 2016). Our data confirm that measurements taken before 60 min do not accurately represent the true purity, but closely correspond to measurements taken at the same time point using phosphor imaging. The purities assessed at later time points are virtually identical by both radioanalytical methods (Additional file 1: Fig. S9). Consequently, our statistical modeling also applies to γ-spectrometry. This suggests that our results are generalizable to any suitable radioTLC method, such as scintillation counting. These experiments are described in full in the Supporting Information.

Our initial iteration of the statistical modeling assigned equal weights to each measurement. The fit is generally good (Additional file 1: Fig. S3). As the aim of these studies is to arrive at a model that can be used primarily to assess the radiochemical purity of mixtures in the 90–100% range, we explored a model in which measurements in this region were given extra weight. This is because reactions with true purity < 90% will likely require purification prior to administration, meaning that highly accurate quantification of the yield is only necessary for purities > 90%. We therefore assigned a weight of 10 times more to each observation > 90%. The result is a model that fit slightly better in upper extreme, and less well in other regions. The weighted model predicts slightly higher true yields for measurements > 90% (Additional file 1: Table S2), but both models demonstrate similar MAE (Additional file 1: Tables S3). As these predictions may prevent unnecessary purification procedures to be performed and reduce analysis time, we validated our weighted model against an independent data set. Predictive accuracy was extremely high (± 1%) (Additional file 1: Fig. S6). By contrast, accuracy of the non-weighted model was slightly lower (± 3%). This suggests that our choice of predictive statistical model is justified.

The mean average error of our model decreases substantially from 30 min to 2 h. This implies that radiochemical purity can be more accurately quantified by measurements taken at least 2 h after the TLC plate is removed from the mobile phase. Quantification of yield at earlier time points may over- or underestimate the true yield depending on the chelating moiety. This is because of the different affinities that the chelators have for the daughter radionuclides of actinium-225. For reactions performed at 95 °C using DOTA, bismuth-213 present in the reaction mixture will be complexed and contribute to the counts detected in grid section 1 of the TLC plate. At early analysis time points, therefore, the activity of the ^225^Ac-labeled compound may be overestimated. It is likely that chelation of bismuth-213 may increase relative to actinium-225 at lower temperatures due to more rapid labeling kinetics (Song et al. 2011), increasing the degree to which early measurements may be misleading. By contrast, bismuth-213 present in the reaction mixture will be incompletely complexed by macropa at any reaction temperature. Consequently, it will migrate along with any actinium-225 that is not bound to the ligands, leading to an underestimation of radiochemical yield.

## Conclusions

As TAT with actinium-225 continues to demonstrate clinical promise, there is a growing need to standardize QC procedures for ^225^Ac-labeled radiopharmaceuticals for safety and regulatory requirements. A major challenge is the ability to accurately quantify radiochemical purity given the time required for actinium-225 to reach secular equilibrium. We compiled a large dataset of empirically-measured radiolabeling yields using multiple radioligands conjugated to DOTA, derivatives of DOTA, or macropa by collecting measurements at various times after running a TLC. Our generalized model confirms that predictive accuracy improves after 30 min and is comparable at all time points from 2 to 6 h. On this basis we argue that the 2 h analysis time point best balances the need to accurately assess the purity of the radiopharmaceutical with the need to release it for administration as quickly as possible.

## Supplementary Information

**Additional file 1.** Further details on the methodology used in this work are provided in this file including Information on precursors, predictions for non-weighted statistical models, validation of weighted statistical models, radio TLC methodology, physical decay models, comparisons of RCP determination by method and certificate of analysis of Actinium -225.

## Data Availability

All data generated during the experiments is reported in the manuscript. Raw data is available from the authors upon request.

## Abbreviations

3D: Three dimensional
3*p*-C-DEPA: 2-[(Carboxymethyl)][5-(4-nitrophenyl-1-[4,7,10-tris(carboxymethyl)-1,4,7,10-tetraazacyclododecan-1-yl]pentan-2-yl)amino]acetic acid
AUC: Area under the curve
CI: Confidence interval
DMSO: Dimethylsulfoxide
DOTA: 1,4,7,10-Tetraazacyclododecane-1,4,7,10-tetracetic acid
EDTA: Ethylenediaminetetraacetic acid
Macropa: *N,N*′-Bis[(6-carboxy-2-pyridyl)methyl]-4,13-diaza-18-crown-6
MAE: Mean average error
MeOH: Methanol
OAc: Acetate
PSMA: Prostate-specific membrane antigen
QC: Quality control
RCP: Radiochemical purity
RCY: Radiochemical yield
R_f_: Retention factor
ROC: Receiver operating characteristics
TAT: Targeted alpha-particle therapy
TLC: Thin layer chromatography
