# Supplementary material for: A suitable time point for quantifying the radiochemical purity of 225Ac-labeled radiopharmaceuticals
Source: EJNMMI Radiopharm Chem. 2021 Dec 20;6:38. doi: 10.1186/s41181-021-00151-y (PMC8688611; doi:10.1186/s41181-021-00151-y)
Supplement: Supplementary file 1 — Additional file 1. Further details on the methodology used in this work are provided in this file including Information on precursors, predictions for non-weighted statistical models, validation of weighted statistical models, radio TLC methodology, physical decay models, comparisons of RCP determination by method and certificate of analysis of Actinium -225. [file 41181_2021_151_MOESM1_ESM.docx]

**Supporting Information**

**A Suitable Time Point for Quantifying the Radiochemical Purity of ^225^Ac-Labeled Radiopharmaceuticals**

James M. Kelly, Alejandro Amor-Coarasa, Elizabeth Sweeney, Justin J. Wilson, Patrick W. Causey, John W. Babich

**Table of Contents**

**1. Precursors for ^225^Ac Labeling**…………………………………………………………………………………..……………………………2

**2. Validation of TLC Method**…………………………………………………………………………………………………………..……….3

**3. Predictions of Non-Weighted Statistical Model**………………………………………….……………………………………….4

**4. Validation of Weighted Statistical Model with an Independent Data Set**………………………………………..…8

**5. RadioTLCs Visualized by Phosphor Imaging**………………………………………………………………………………..……….9

**6. Distribution of Measurements by Range**……………………………………………………………………………………………10

**7. Comparison of RCP Determined by Different Methods**…………………………………………………………………….11

**8. Certificate of Analysis of Ac-225**……………………………………………………………………………………………….……….15

1. **Compounds Used in This Study**


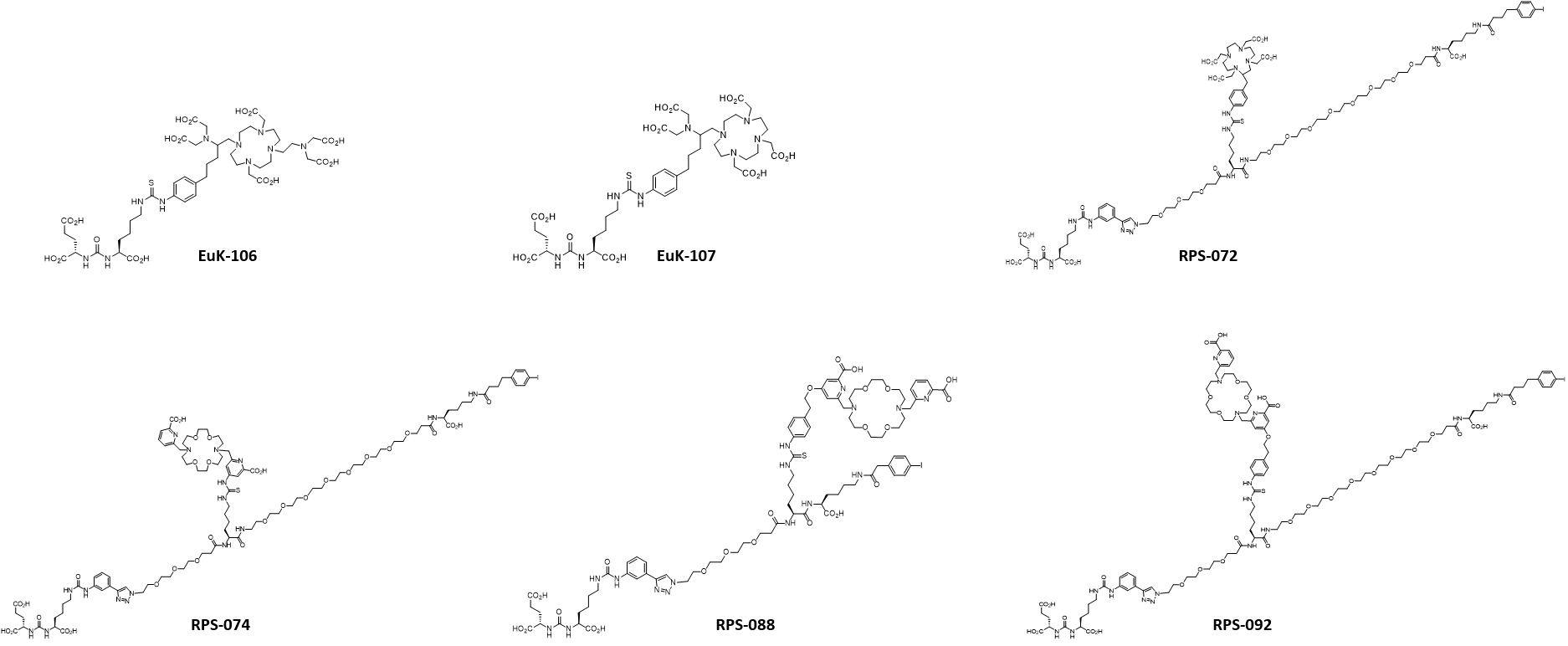


**Figure S1.** Precursors for ^225^Ac labeling.

1. **Validation of TLC Method**

**
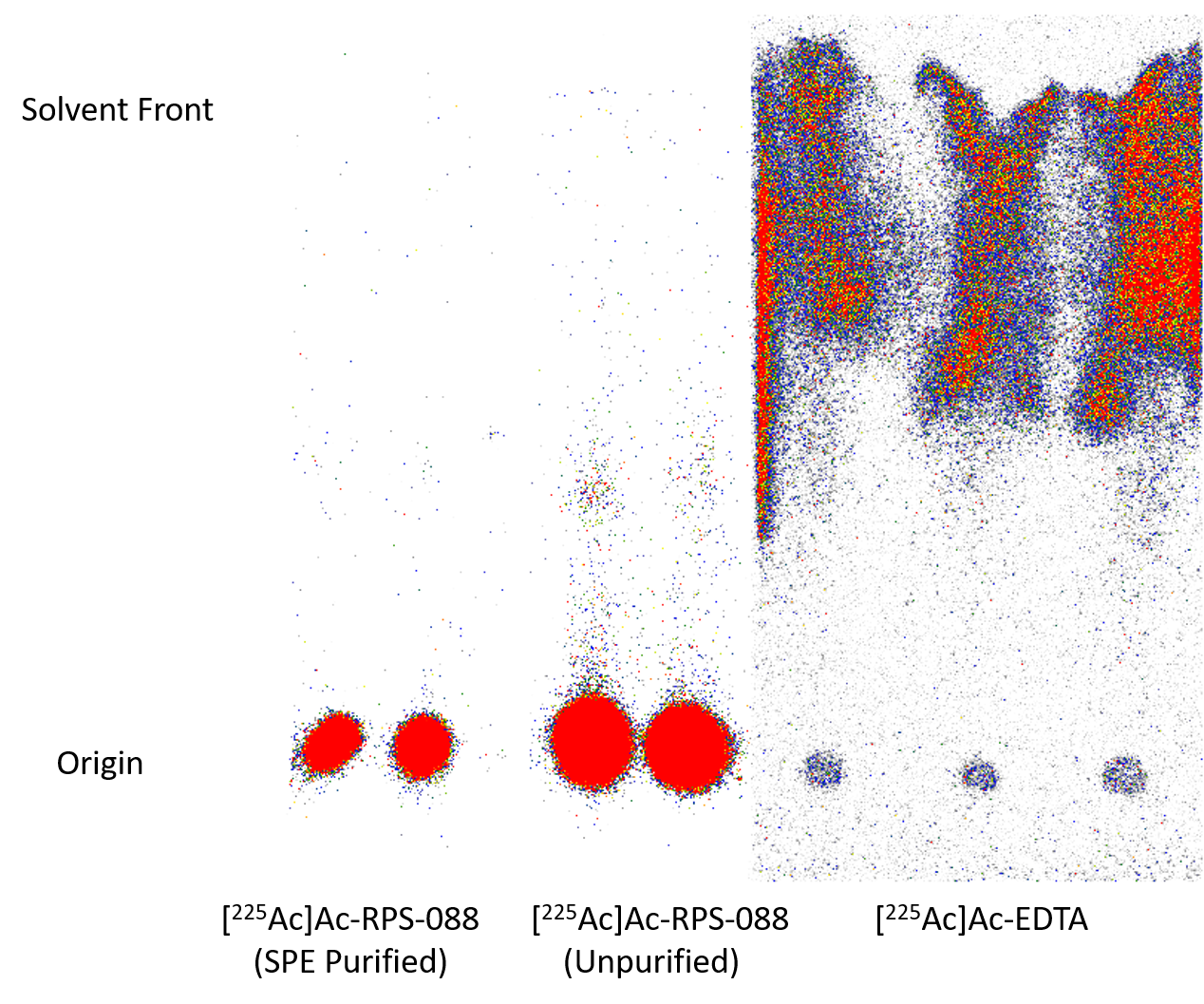
**

**Figure S2.** Comparison of phosphor images of the purified [^225^Ac]Ac-RPS-088 reaction (duplicate; left panel), the same [^225^Ac]Ac-RPS-088 reaction pre-purification (duplicate; middle panel), and [^225^Ac]Ac-EDTA (triplicate; left panel). TLC plates were run in a 10% *v/v* MeOH/10 mM aqueous EDTA mobile phase, and measurements were taken 2 h after the TLC plate was developed. The labeled ligand does not migrate while the EDTA-complexed metal migrates approximately with the solvent front.

1. **Non-Weighted Statistical Model**


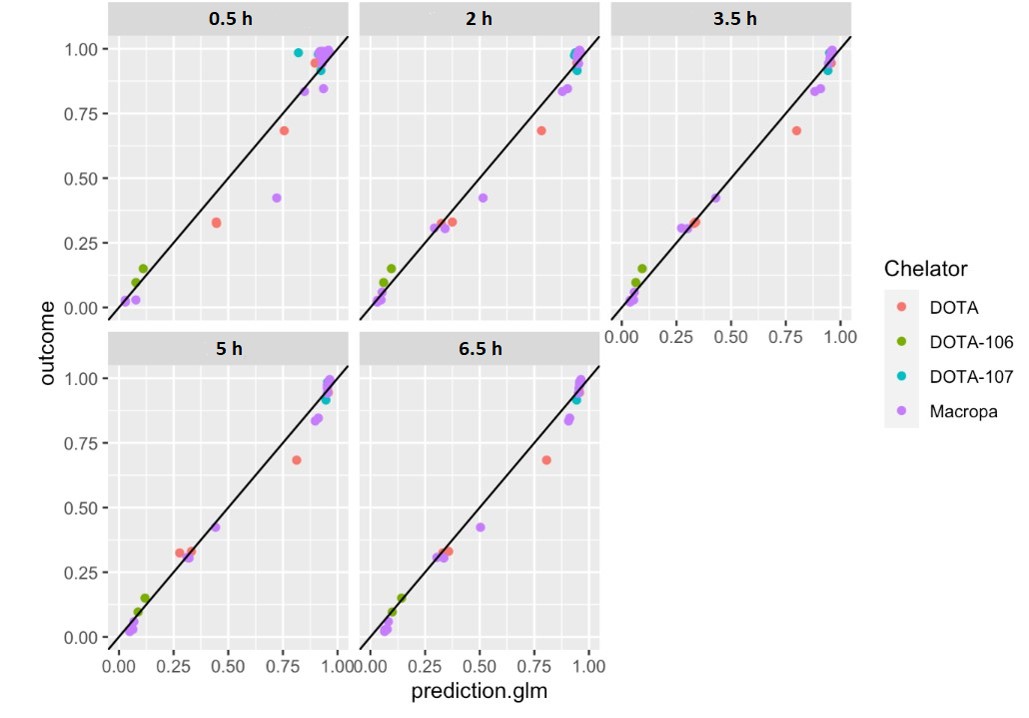


**Figure S3**. Prediction of labeling yield using a training set in which all observations are assigned an equal weight.

| **RCY AT 0.5 h (%)** | **EXPECTED RCY (%)** | **RCY at 2.0 h (%)** | **EXPECTED RCY (%)** | **RCY at 3.5 h (%)** | **EXPECTED RCY (%)** | **RCY at 5.0 h (%)** | **EXPECTED RCY (%)** | **RCY at 6.5 h (%)** | **EXPECTED RCY (%)** |
| --- | --- | --- | --- | --- | --- | --- | --- | --- | --- |
| **0** | 2 (0,7) | **0** | 2 (1,7) | **0** | 3 (2,7) | **0** | 4 (2,9) | **0** | 6 (2,16) |
| **10** | 4 (1,12) | **10** | 5 (2,11) | **10** | 6 (3,11) | **10** | 8 (4,15) | **10** | 10 (4,23) |
| **20** | 8 (3,20) | **20** | 10 (5,18) | **20** | 12 (7,19) | **20** | 15 (9,23) | **20** | 18 (9,33) |
| **30** | 15 (7,30) | **30** | 18 (11,29) | **30** | 21 (14,30) | **30** | 25 (17,35) | **30** | 28 (16,44) |
| **40** | 28 (16,44) | **40** | 31 (21,43) | **40** | 35 (26,44) | **40** | 38 (29,49) | **40** | 42 (28,58) |
| **50** | 45 (30,61) | **50** | 48 (37,59) | **50** | 51 (42,60) | **50** | 54 (43,65) | **50** | 57 (42,71) |
| **60** | 64 (48,77) | **60** | 66 (55,75) | **60** | 68 (59,75) | **60** | 69 (59,78) | **60** | 71 (56,83) |
| **70** | 79 (65,88) | **70** | 80 (70,87) | **70** | 80 (73,86) | **70** | 81 (72,88) | **70** | 82 (68,91) |
| **80** | 89 (77,95) | **80** | 89 (82,94) | **80** | 89 (83,93) | **80** | 89 (81,94) | **80** | 89 (77,95) |
| **90** | 94 (86,98) | **90** | 94 (89,97) | **90** | 94 (90,97) | **90** | 94 (88,97) | **90** | 94 (84,98) |
| **91** | 95 (87,98) | **91** | 95 (90,97) | **91** | 95 (90,97) | **91** | 94 (89,97) | **91** | 94 (85,98) |
| **92** | 95 (87,98) | **92** | 95 (90,98) | **92** | 95 (91,97) | **92** | 95 (89,98) | **92** | 95 (86,98) |
| **93** | 96 (88,98) | **93** | 95 (91,98) | **93** | 95 (91,97) | **93** | 95 (90,98) | **93** | 95 (86,98) |
| **94** | 96 (89,99) | **94** | 96 (91,98) | **94** | 96 (92,98) | **94** | 95 (90,98) | **94** | 95 (87,98) |
| **95** | 96 (89,99) | **95** | 96 (92,98) | **95** | 96 (92,98) | **95** | 96 (91,98) | **95** | 95 (87,98) |
| **96** | 96 (90,99) | **96** | 96 (92,98) | **96** | 96 (93,98) | **96** | 96 (91,98) | **96** | 96 (88,99) |
| **97** | 97 (90,99) | **97** | 96 (92,98) | **97** | 96 (93,98) | **97** | 96 (92,98) | **97** | 96 (88,99) |
| **98** | 97 (91,99) | **98** | 97 (93,99) | **98** | 97 (93,98) | **98** | 96 (92,98) | **98** | 96 (89,99) |
| **99** | 97 (91,99) | **99** | 97 (93,99) | **99** | 97 (94,98) | **99** | 97 (92,99) | **99** | 96 (89,99) |
| **100** | 97 (92,99) | **100** | 97 (94,99) | **100** | 97 (94,98) | **100** | 97 (93,99) | **100** | 97 (89,99) |

**Table S1.** Prediction of true labeling yield, defined as the value measured at 26 h post-TLC, on the basis of measurements made at either 0.5 h, 2 h, 3.5 h, 5 h, or 6.5 h post-TLC.

|  | **All** | **Macropa** | **DOTA** |
| --- | --- | --- | --- |
| *Time (h)* | *Mean absolute error* | *Mean absolute error* | *Mean absolute error* |
| 0.5 | 0.059 | 0.047 | 0.023 |
| 2 | 0.032 | 0.049 | 0.036 |
| 3.5 | 0.027 | 0.051 | 0.035 |
| 5 | 0.028 | 0.052 | 0.023 |
| 6.5 | 0.030 | 0.043 | 0.021 |

**Table S2.** Mean absolute error (MAE) of predictions made using a model that assigned equal weight to each observation. MAE was calculated by taking the average absolute value of the difference between each prediction and unity. Predictions were made for a model constructed using data from all ligands, the ligands containing the macropa chelator, and the compounds containing DOTA or a DOTA-derivative.


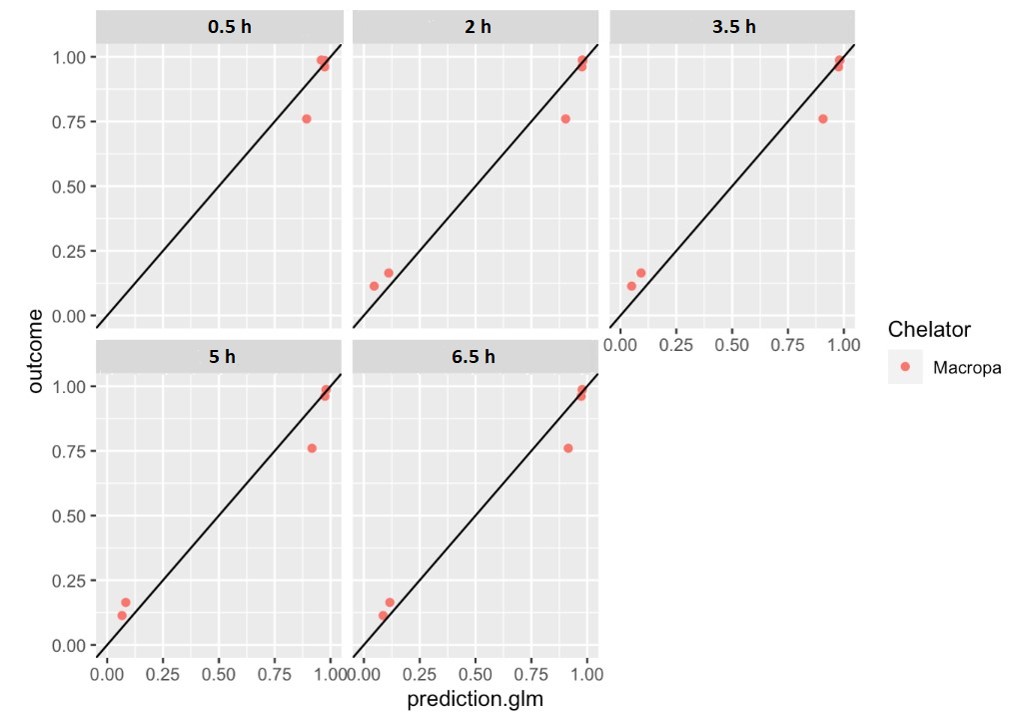


**Figure S4**. Prediction of labeling yield using a training set derived from macropa-conjugated ligands only.


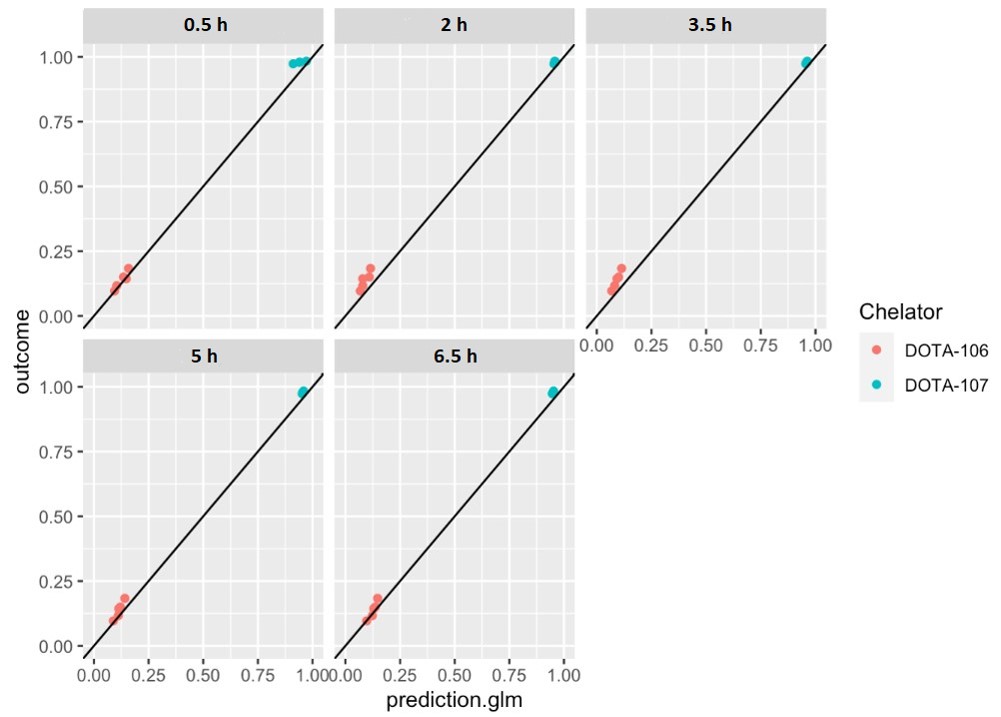


**Figure S5.** Prediction of labeling yield using a training set derived from DOTA-conjugated ligands only. All DOTA measurements randomly assigned to the training set, meaning that the validation set contains DOTA-106 and DOTA-107 predictions only.

1. **Validation of the Weighted Statistical Model**

**
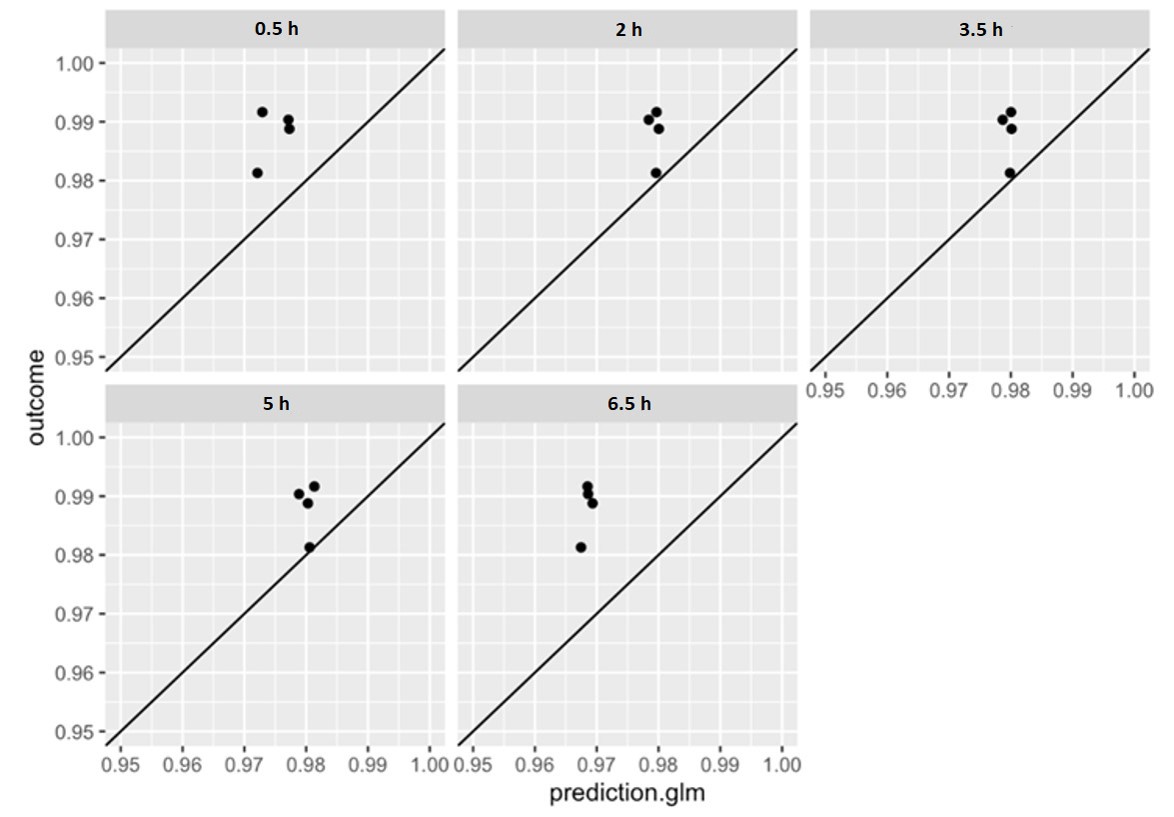
**

**Figure S6.** Predicted yield versus true yield for an independent data set collected by labeling a macropa-derived antibody with ^225^Ac. Predictions were made using the weighted model.

| *Time (h)* | *Mean absolute error* |
| --- | --- |
| 0.5 | 0.013 |
| 1.0 | 0.009 |
| 1.5 | 0.008 |
| 2.0 | 0.008 |
| 8.0 | 0.020 |

**Table S3.** Mean average error of the predictions.

1. **RadioTLCs Visualized by Phosphor Imaging**

**
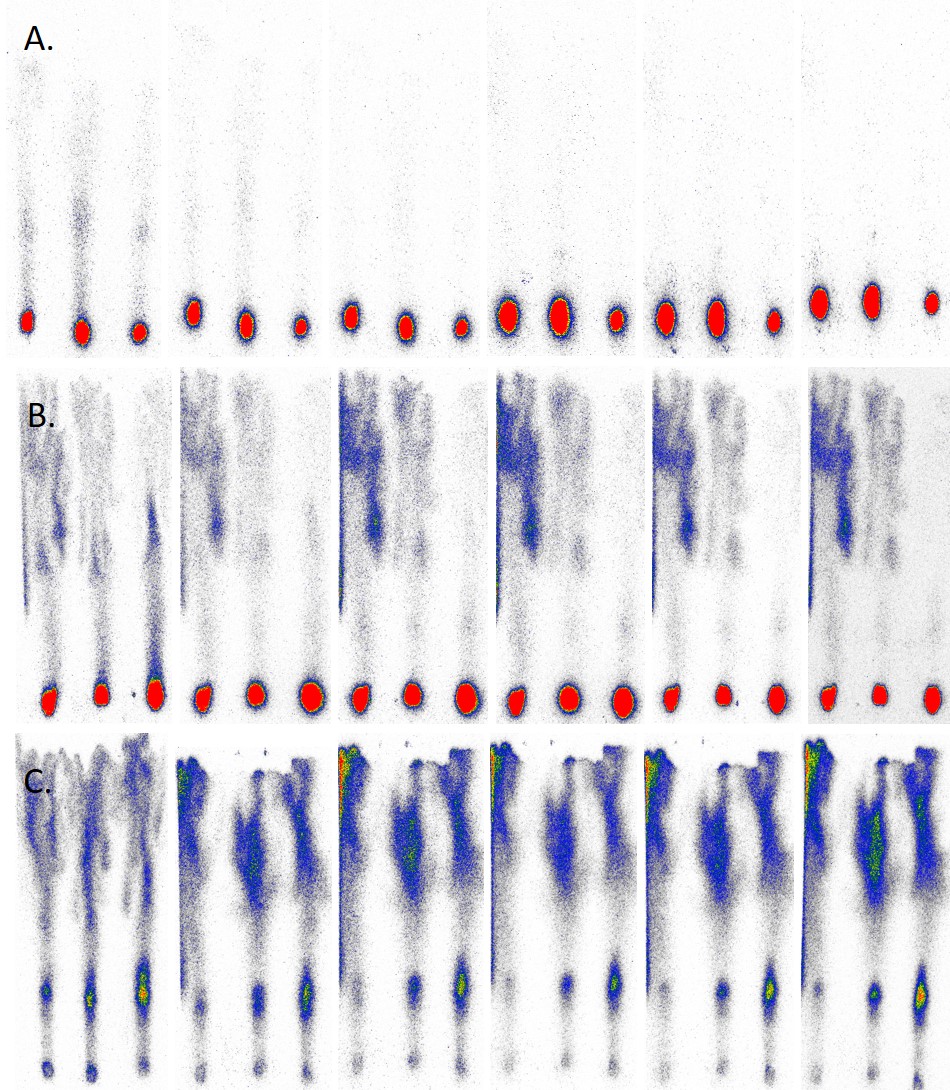
**

1. **Distribution of Measurements by Range**


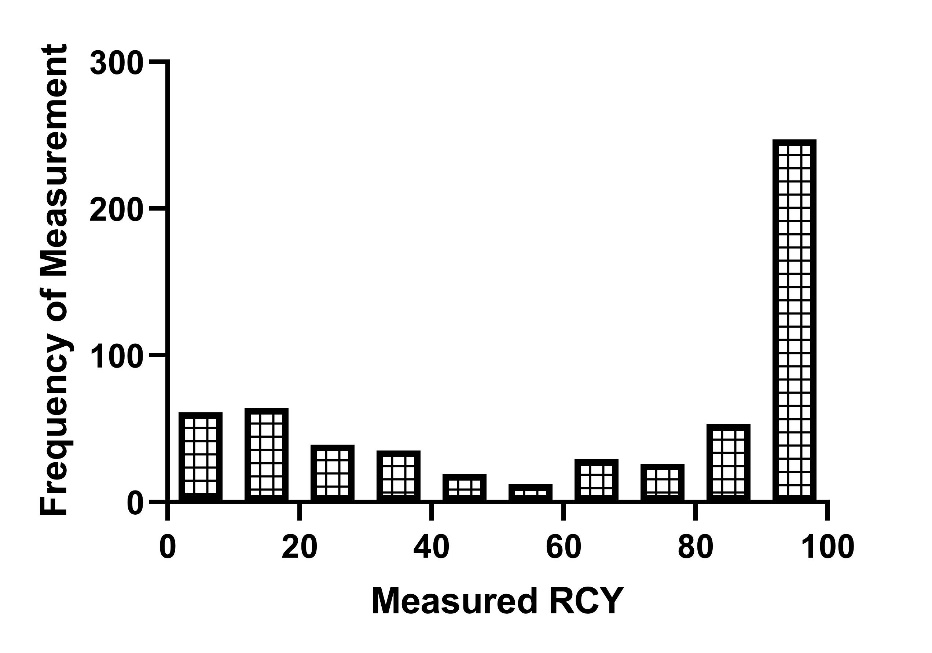


**Figure S8.** Distribution of measurements. Measurements are grouped in ranges of 10, i.e. 0-10%, 10-20%, etc.

1. **Comparison of RCP as Assessed by Phosphor Imaging and NaI(**[***1***](#_ENREF_1)**) Scintillation Detection**

*Analysis of Reaction Yield: NaI(*[*1*](#_ENREF_1)*) Scintillation Detector*

Three model systems were prepared by incubation of 1 mL solutions of 205 kBq in 1 M NH_4_OAc with either 18.2 µM EuK-106 for 1 min at 95 °C, 9.0 µM RPS-072 for 5 min at 95 °C, or 10.4 µM RPS-088 for 15 min at 95 °C. Each reaction was analyzed in triplicate by depositing 3 µL aliquots of the reaction mixture on the origin of a silica gel 60-coated aluminum plate (Sigma Aldrich). The plates were run in 10% *v/v* MeOH/10 mM EDTA (in H_2_O) as described above. The plates were then cut into three sections and the sections were counted separately using a NaI([*1*](#_ENREF_1)) scintillation detector with multi-channel analyzer (Canberra). Regions-of-interest were defined from 190-250 keV and 400-480 keV to quantify the contributions of ^221^Fr (γ = 218 keV) and ^213^Bi (γ = 440 keV). RCP was calculated by determining the ratio of counts in the 190-250 keV region in the section containing the product to the sum of the counts in the 190-250 keV regions on all three sections.

We compared the RCP as assessed by phosphor imaging to the purity as determined using NaI([*1*](#_ENREF_1)) scintillation detection. We selected as representative examples for comparison one reaction resulting in a low (< 25%) final product yield, one reaction with intermediate yield (25% < RCY < 75%), and one reaction with a high (> 90%) yield. The purities as assessed by the two methods closely agree (Figure S9).


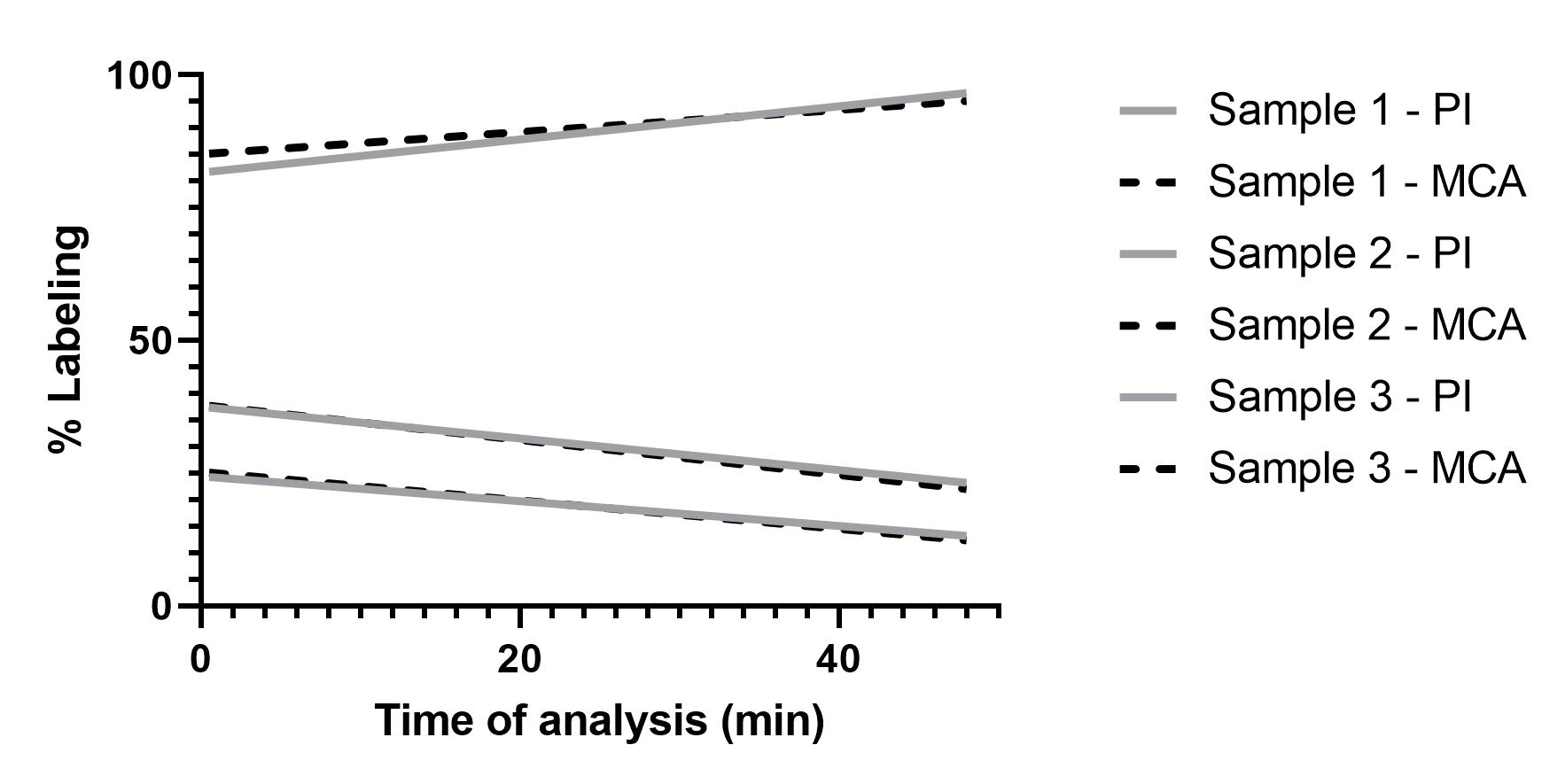


**Figure S9.** RCP of three reactions determined by either phosphor imaging (solid gray line) or scintillation detection with multichannel analyzer (dashed black line). The TLC plates were analyzed from 0.5 h to 48 h after running in a 10% MeOH/10 mM EDTA mobile phase.

On the basis of these results, we suggest that the mathematical modeling developed using the phosphor imaging data is also applicable to assessment of RCP by NaI(Tl) scintillation detection with a multichannel analyzer. Consequently, the recommended time of analysis is also 2 h after removing the TLC plate from the mobile phase.


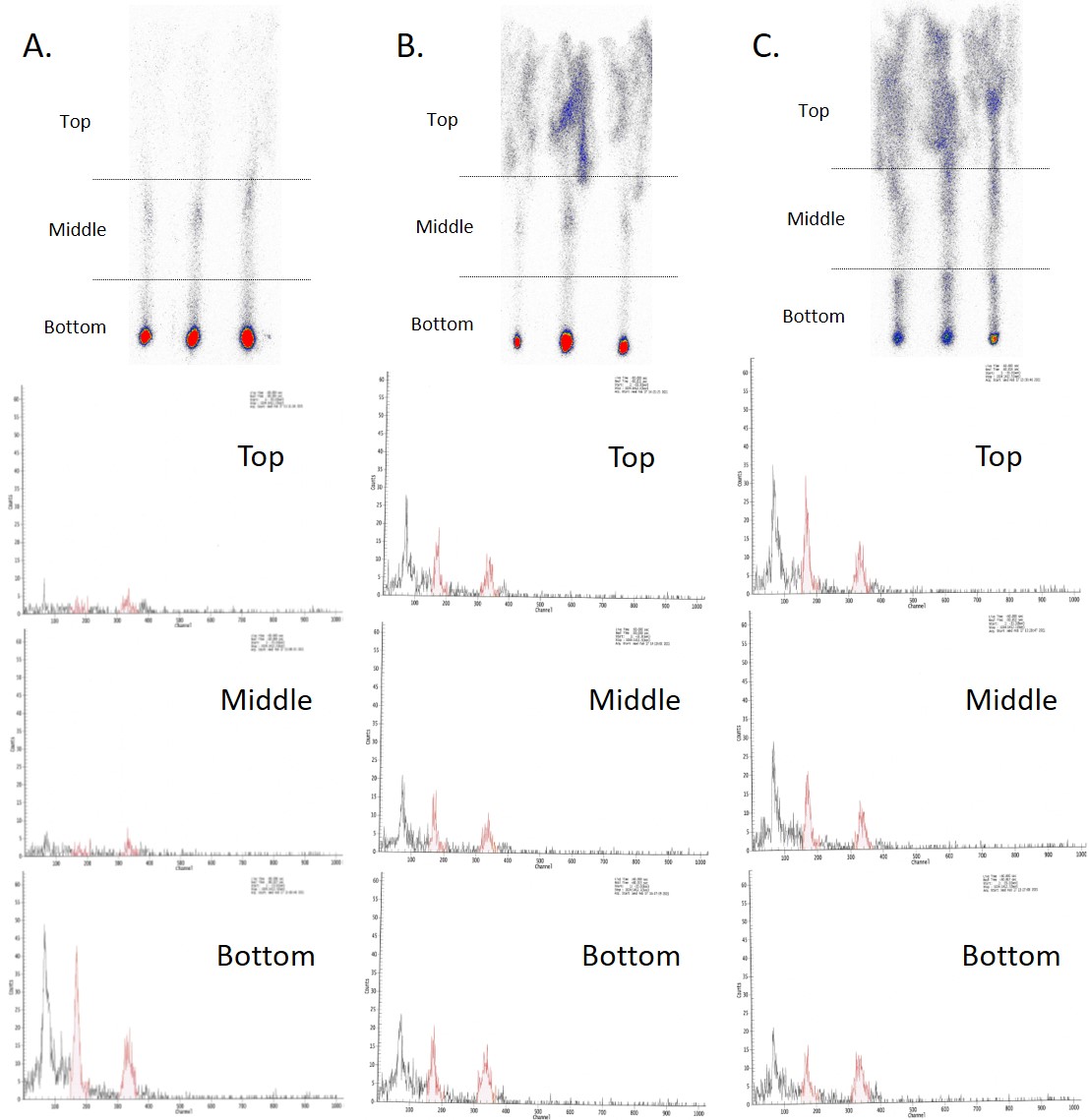


**Figure S10.** TLC plates with their corresponding MCA spectra. Both analyses were performed 30 min after the TLC plate was removed from the mobile phase. The sections into which the TLC plates were cut for γ-spectrometry are indicated. Regions-of-interest (190-250 keV and 400-480 keV) are highlighted in red. Each column corresponds to one compound. A. [^225^Ac]RPS-088. B. [^225^Ac]Ac-RPS-072. C. [^225^Ac]Ac-EuK-106.

High resolution gamma spectroscopy was also used to assess RCP. TLC plates were prepared and run as described above and immediately cut into three sections. Beginning 15 min after the TLC plates were removed from the mobile phase, each section was counted separately for 10 min in a Ge(Li) detector. The total time required to count each TLC strip was 33 min. RCP was defined as the ratio of the counts due to the 218 keV peak in the bottom section to the total number of counts due to the 218 keV peaks. The purity of the [^225^Ac]Ac-RPS-088 was determined to be > 99% (Figure S11) at 1 h after removing the TLC plate from the mobile phase, the time at which all three TLC sections had been counted. The purity of the [^225^Ac]Ac-RPS-088 was determined to be 10.6 ± 1.5% (Figure S11).


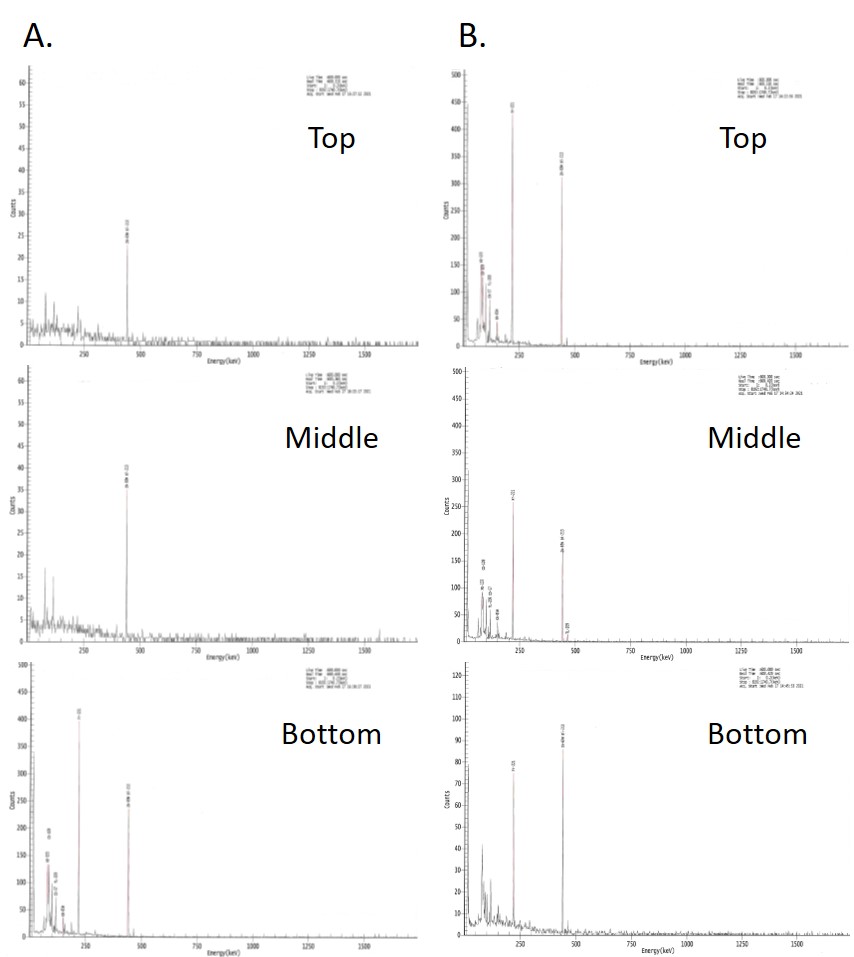


**Figure S11**. High resolution γ-spectroscopic determination of RCP using a Ge(Li) detector. A. [^225^Ac]Ac-RPS-088. B. [^225^Ac]Ac-EuK-106.

By comparison, the true purities, as assessed by phosphor imaging at 24 h, of these two reactions were 96.6 ± 1.4% and 13.2 ± 0.6%, respectively. We therefore propose that high resolution gamma spectroscopy can be also be used to determine RCP prior to batch release. However, due to the geometry of the detector, it is necessary to count samples for at least 10 min in order for sufficient counts to be detected. Even under these circumstances, counts due to the 218 keV peak may be below the limit of detection. While this is indicative of very low activities of francium-221 (and by inference, actinium-225), it may be problematic in borderline cases, such as products whose radiochemical purity is determined to be 94-96%.

1. **Certificate of Analysis for Ac-225**
